# Kynurenic acid blunts A1 astrocyte activation against neurodegeneration in HIV-associated neurocognitive disorders

**DOI:** 10.1186/s12974-023-02771-4

**Published:** 2023-03-30

**Authors:** Jingxian Lun, Yubin Li, Xuefeng Gao, Zelong Gong, Xiaoliang Chen, Jinhu Zou, Chengxing Zhou, Yuanyuan Huang, Bingliang Zhou, Pengwei Huang, Hong Cao

**Affiliations:** Department of Microbiology, Guangdong Provincial Key Laboratory of Tropical Disease Research, School of Public Health, Southern Medical University, Guangzhou, 510515 Guangdong China

**Keywords:** Kynurenic acid, HIV-1 gp120, Neurotoxic astrocytes, α7nAChR, Tryptophan

## Abstract

Despite extensive astrocyte activation in patients suffering from HIV-associated neurocognitive disorders (HAND), little is known about the contribution of astrocytes to HAND neuropathology. Here, we report that the robust activation of neurotoxic astrocytes (A1 astrocytes) in the CNS promotes neuron damage and cognitive deficits in HIV-1 gp120 transgenic mice. Notably, knockout of α7 nicotinic acetylcholine receptors (α7nAChR) blunted A1 astrocyte responses, ultimately facilitating neuronal and cognitive improvement in the gp120tg mice. Furthermore, we provide evidence that Kynurenic acid (KYNA), a tryptophan metabolite with α7nAChR inhibitory properties, attenuates gp120-induced A1 astrocyte formation through the blockade of α7nAChR/JAK2/STAT3 signaling activation. Meanwhile, compared with gp120tg mice, mice fed with tryptophan showed dramatic improvement in cognitive performance, which was related to the inhibition of A1 astrocyte responses. These initial and determinant findings mark a turning point in our understanding of the role of α7nAChR in gp120-mediated A1 astrocyte activation, opening up new opportunities to control neurotoxic astrocyte generation through KYNA and tryptophan administration.

**Supplementary Information:**

The online version contains supplementary material available at 10.1186/s12974-023-02771-4.

## Introduction

Despite tremendous progress in combination with antiretroviral therapy, HIV-associated neurocognitive disorders (HAND) remain a common complication in HIV-1 infected patients, especially those with improved life expectancies [1–3]. One possible explanation underlying the pathogenicity of HAND is the sustained immune activation and cytotoxic effects produced by the critical HIV-1 glycoprotein 120 (gp120), leading to a chronic inflammation scenario in the CNS [4, 5]. Astrocytes, the most abundant glial cells in the CNS, are functionally indispensable for maintaining neuronal survival, synaptic functions, and the blood–brain barrier [6]. The heterogeneous population of reactive astrocytes can turn into a detrimental (A1 astrocytes) or neuroprotective phenotype (A2 astrocytes) in a diseased context [7–9]. Beyond the discrepant terminology, whether the A1 astrocytes in the several disorder onset and progression also holds for gp120-triggered neurodegeneration is needed to be directly addressed [10].

Kynurenic acid (KYNA), the end-stage product of tryptophan metabolism, is primarily synthesized and liberated by cerebral astrocytes [11–13]. Later studies confirmed that endogenous KYNA acts as a non-competitive antagonist of α7 nicotinic acetylcholine receptors (α7nAChR) at physiological concentrations [14]. α7nAChR is abundant in the hippocampus, notably in astrocytes, participating in neuronal synaptic transmission, memory, and cognition processes [15–17]. Additionally, α7nAChR stimulation recruits Janus kinases 2 (JAK2)/signal transducer and activator transcription 3 (STAT3), a canonical molecule controlling reactive transformation in astrocytes [18, 19]. Given that α7nAChR activation leads to the recruitment of JAK2/STAT3 to its receptor complex, inhibition of α7nAChR through KYNA may offer a plausible approach to modulate the formation of reactive astrocytes upon gp120 insult.

Here, we took advantage of primary astrocyte cultures, gp120 transgenic mice (gp120tg mice), α7nAChR knockout mice (α7^−/−^ mice), and α7^−/−^gp120tg mice and aimed to investigate the effect of KYNA in gp120-induced astrocytes. We tested the hypothesis that α7nAChR-mediated JAK2/STAT3 signaling inhibition might block gp120-driven neurotoxic astrocyte activation. Finally, we examined whether tryptophan, a source of endogenous KYNA, could improve neuronal deficits in gp120tg mice by regulating neurotoxic astrocytes.

## Materials and methods

### Primary cell culture and drug treatments

Primary cultures of astrocytes from the rat cortex were established and maintained as previously published protocol [20]. Briefly, cortical astrocytes were dissected from Sprague–Dawley (SD) neonatal rats (1–3 days) with the aseptic operation and digested with 0.25% trypsin (Gibco, USA) at 37 °C for 20 min. The cell suspension was filtered through the cell strainer (75 μm pore size, Bioland, China) and seeded into the poly-d-lysine coated T-75 flasks (Corning, USA) with DMEM/F12 (Gibco, USA) supplemented with 10% FBS, 1% penicillin/streptomycin. After 10 days of incubation, the cells were purified in a shaking incubator (250 r/min) at 37 °C for 24 h, and astrocytes were cultured at desired densities in 6-well plates. The cells were identified as > 98% pure astrocytes by Cy3-conjugated GFAP immunostaining (at 1:400 dilution, Abcam, UK, ab49874). Cultured astrocytes were treated with 150 pM HIV-1 gp120 CM (ProSpec-Tany TechnoGene Ltd., Israel) for 12 h before application of 5 or 25 μM KYNA (Sigma-Aldrich, USA) for a further 12 h. In addition, gp120-induced (150 pM, 11 h) astrocytes were pretreated for 1 h with media in the presence or absence of 10 nM methyllycacontitine (MLA, MCE, USA) or 50 μM AG490 (Selleckchem, USA) followed by a 12 h co-incubation period with KYNA.

### Total RNA isolation and quantitative reverse transcription PCR (RT-qPCR) analysis

According to the manufacturer’s instructions, total RNA was extracted from samples using RNAiso Plus Reagent. Reverse transcription was performed following the protocol of the PrimeScript™ RT Master Mix kit. Then, cDNA was run for quantitative PCR using TB Green® Premix Ex Taq™ II (Tli RNaseH Plus) kit on QuantStudio 6 and 7 Flex real-time PCR systems (Thermo Fisher, USA). The reagents mentioned above were obtained from Takara (Japan). Primers for RT-qPCR were provided by Sangon Co. (Shanghai, China) and attached to Additional file 1: Tables S1, S2. The extracellular or plasma levels of Il-1β, Il-6, and Tnf-α were confirmed by ELISA using commercially available kits.

### Western blotting

Clarified lysates were fractionated by SDS-PAGE using 8–15% gradient polyacrylamide gels and transferred onto a PVDF membrane. After blocking with TBST containing 5% non-fat milk (Bio-Rad, USA) for 1 h, the membranes were stained with primary antibodies in the diluted solution overnight at 4 °C. Primary antibodies used were: anti-MAP2 (1:1000, 17490-1-AP), anti-NeuN (1:1000, 26975-1-AP), anti-Synaptophysin (1:5000, 17785-1-AP), anti-GFAP (1:5000, 16825-1-AP), anti-C3/C3b/C3c (1:1000, 21337-1-AP), anti-CHRNA7 (1:2500, 21379-1-AP), anti-JAK2 (1:500, 17670-1-AP), anti-STAT3 (1:2000, 10253-2-AP), rabbit monoclonal anti-phosphorylated-JAK2 (1:5000, phospho Y1007 + Y1008, Abcam, UK, ab32101), rabbit monoclonal anti-phosphorylated-STAT3 (1:10,000, phospho Y705, Abcam, UK, ab267373), and mouse monoclonal anti-GAPDH (1:10,000, 17670-1-AP). Except for the indicated antibodies, other antibodies listed above were rabbit polyclonal antibodies purchased from Proteintech (USA). Following incubation with appropriate HRP-conjugated goat anti-rabbit IgG secondary antibodies (1:5000, Bioss, USA, bs-0295G-HRP) or HRP-conjugated goat anti-mouse IgG secondary antibodies (1:5000, Bioss, USA, bs-0296G-HRP) for 1 h at room temperature, the membrane was treated with Clarity Western ECL blotting substrate (Bio-Rad, 1705060, USA). Bands densitometry were quantified using Fuji ImageJ software (Additional file 2).

### Immunolabeling assay for primary astrocytes

Cells were fixed with 100% methanol for 20 min at − 20 °C and permeabilized with 0.25% Triton X-100 prepared in PBS for 10 min, followed by blockage with mixture buffer (1% w/v BSA, Sigma-Aldrich, USA; 22.52 mg/ml glycine in PBST). The cells were incubated with mouse monoclonal Cy3-conjugated anti-GFAP (1:400, not requiring secondary antibody, Abcam, UK, ab49874), anti-C3/C3b/C3c (1:500, 21337-1-AP), Alexa Fluor 488-conjugated α-bungarotoxin (5 μg/ml, Thermo Fisher Invitrogen, USA, B13422) for overnight at 4 °C, followed by incubation with Alexa Fluor 488-conjugated goat anti-rabbit secondary antibody (1:500, Abcam, UK, ab150077), goat anti-rabbit IgG H&L (Cy5, 1:2500, Abcam, UK, ab6564) for 2 h in the dark at room temperature. DAPI (4 μg/ml, Thermo Fisher, USA, 62248) was used as nuclei stain. Stained cells were examined using a fluorescent microscope (E800 Nikon, Japan) connected to a color digital camera.

### Animals and treatment

The HIV-1 gp120 transgenic mice (gp120tg mice, 3 months old, 6 months old, 12 months old, 24 months old; *n* = 20 per group; 10 females and 10 males) on SJL/BL6/129 background (cross between C57BL/6J female × Sv129 male) expressed gp120 in astrocytes under the control of a modified murine *Gfap* gene. The α7nAChR knockout mice (α7^−/−^ mice, B6.129S7-Chrna7tm1Bay/J) were purchased from the Jackson Laboratory. The gp120tg mice intercrossed with α7^−/−^ mice to generate α7^−/−^gp120tg mice. C57BL/6J black mice (12 months old) were used as wild-type controls. Genotypes of all mice were confirmed by PCR analysis of tail DNA with the following primer sequences: gp120 forward, 5′-GCGGGAGAATGATAATGGAG-3′; gp120 reverse, 5′-TATGGGAATTGGCTCAAAGG-3′; α7nAChR forward, 5′-TTCCTGGTCCTGCTGTGTTA-3′; α7nAChR wild-type reverse, 5′-ATCAGATGTTGCTGGCATGA-3′; α7nAChR knockout reverse, 5′-CCCTTTATAGATTCGCCCTTG-3′. All mice were bred in groups of 5 mice and maintained in a pathogen-free animal facility with free access to autoclaved distilled water and standard chow under a constant temperature of 22 ± 1 °C on a 12 h light/dark cycle. For tryptophan (Trp) supplement, 12-month-old mice were randomly divided into 4 groups using a random number generator (GraphPad), including WT + Vehicle, WT + Trp, gp120tg + Vehicle, gp120tg + Trp (*n* = 20 mice per group; 10 females and 10 males). Mice were fed the control diet supplemented with the 0.1% tryptophan (Sigma-Aldrich, USA) dissolved in sterile saline (0.9% NaCl) for 1 month.

### Morris water maze test and open field test

For the MWM test, mice used 4 unique geometric figures providing landmarks in the testing room to locate a submerged platform (1 cm below the white-opaque water surface) in a circular pool (1.2 m in diameter and 76 cm in height). The mice were allowed 60 s to locate the hidden platform in one maze quadrant from a randomized starting position for 4 trails per day over 5 consecutive days. Upon completion of training, the mice were allowed to locate the removed platform for 60 s in the retrieval test. The performances were recorded with a camera suspended 250 cm above the center and analyzed by the image tracking system. Mice were also evaluated in the open field test for anxiety and exploratory behavior. Briefly, mice were allowed to move unfetteredly in a brightly lit, square enclosure arena (40 × 40 cm) for 15 min on 2 consecutive days. The distance moved and time spent in the central area were videotaped with a near-infrared camera positioned above the center of the arena. The central zone was 32 cm in diameter with 4 cm from the peripheral walls. An effective center entry was not deemed to have occurred until 90% or over of the mouse’s body had entered the designated center.

### Histopathology assessment

Upon anesthesia of mice with sodium pentobarbital (60 mg/kg, Sigma-Aldrich, USA), brains were dissected and fixed by immersion in 10% formalin solution overnight at room temperature. To view cellular and tissue structure in detailed, deparaffinized and rehydrated slides were counterstained with Mayers Hematoxylin and alcoholic-Eosin (H&E). In addition, sections were labeled with 0.1% cresyl violet solution at 37 °C for 10 min after deparaffinization and hydration for Nissl staining. Next, the slides were differentiated in 95% ethyl alcohol for 5 to 10 min, followed by incubation with 100% alcohol and xylene.

### Immunofluorescence and immunohistochemistry

For immunofluorescence, deparaffinized and rehydrated brain sections were stained with anti-MAP2 (1:500), anti-NeuN (1:500), Cy3-conjugated anti-GFAP (1:400), and anti-C3/C3b/C3c (1:500). Following PBS washes, slides were reacted with Alexa Fluor 488-labeled goat anti-rabbit antibody (1:1000) at room temperature for 1 h. The slides were washed and subsequently counterstained with DAPI (1.5 μg/ml, Thermo Fisher, USA). Five sections from the hippocampus were randomly selected, and the numbers of C3^+^/GFAP^+^ cells were counted. For immunohistochemistry, slides were reacted with anti-phosphorylated-JAK2 (1:100) and anti-phosphorylated-STAT3 (1:100) antibodies overnight at 4 °C in a humidity chamber. After rinsing with PBS, sections were stained with a biotinylated antibody for 60 min and then incubated with 3,3′-diaminobenzidine tetrahydrochloride (DAB) solution for 5 min. Subsequently, sections were counterstained with hematoxylin.

### Image processing and quantification

For each mouse, three sections were randomly selected. Fluorescent immunostained brain sections were imaged using a confocal microscope. Images were processed by Fiji ImageJ, and the background was subtracted before quantification. For the quantification of GFAP^+^/C3^+^ double-staining cells, GFAP^+^ cells were first chosen as a region of interest (ROI). The mean gray of ROI in the C3 channel was measured to indicate the quantification of GFAP^+^/C3^+^ cells.

### Statistical analysis

Graphs and statistical significance are generated by GraphPad Prism 8 software (version 8.3.0). The distribution of the data was assumed to be normal without formal statistical tests. Data from the Morris water maze were determined by repeated measure ANOVA with Bonferroni’s post hoc tests. Two-group differences were analyzed using an unpaired Student’s *t* test (two-tailed). One-way ANOVA followed by post hoc Tukey’s multiple comparisons was used to compare the differences in multiple groups.

## Results

### Age-related neurological deficits in gp120tg mice

**Fig. 1:**
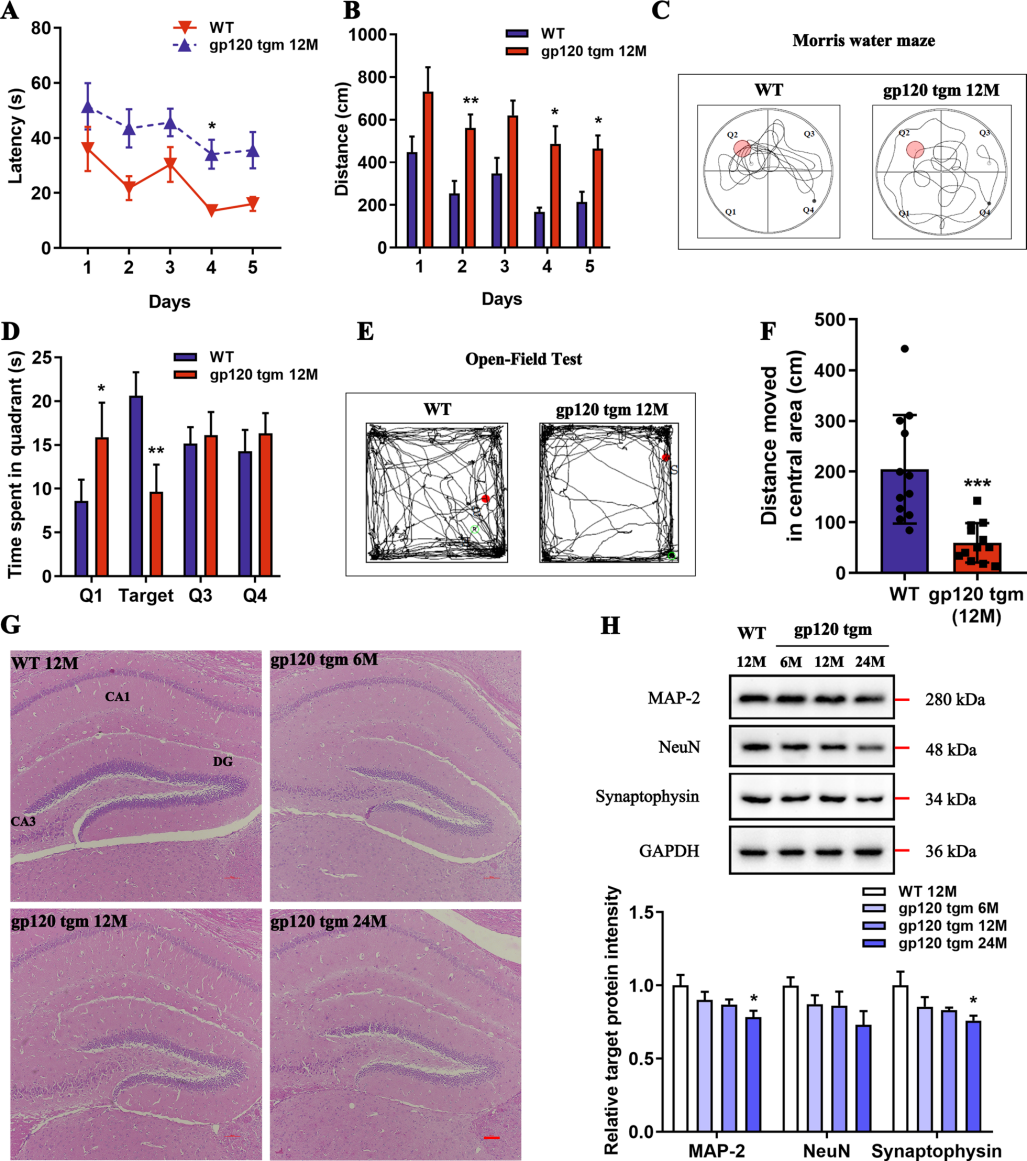
HIV-1 gp120 expression induces progressive neuropathology. **A** Latency and **B** distance to locate the submerged platform during 5 training days in the MWM test (*n* = 12 mice per group, *F*_1, 22_ = 18.09, *P* = 0.0003; *F*_1, 22_ = 58.60, *P* < 0.0001, respectively). **C** Searching strategies for the removed platform in the probe trial. Black dots depict the initial position, and white dots represent the final position. **D** Time spent in the target quadrant in the retrieval test (*F*_1, 8_ = 0.0307, *P* = 0.08653). **E** Representative performance of mice during the open field test. Red dots reflect the starting position, and green dots depict the ending position (*n* = 12 mice per group). **F** Bars show the distance traveled in the central zone (*t* = 4.413, *P* = 0.0002). **G** H&E staining shows the histology of the hippocampus (scale bar: 100 μm, *n* = 3 mice per group). **H** Quantification of MAP-2, NeuN, and Synaptophysin protein levels in the hippocampus (*n* = 4 mice per group, *F*_3,12_ = 2.751, *P* = 0.0888; *F*_3,12_ = 1.967, *P* = 0.1727; and *F*_3, 12_ = 2.745, *P* = 0.0892, respectively). In **A**–**F**, data are presented as mean ± SEM. In **A**–**D**, data are analyzed by repeated measure ANOVA with Bonferroni’s post hoc tests. In **F**, data are analyzed by unpaired Student’s *t* test, two-tailed. In **H**, data are analyzed by One-way ANOVA with Tukey’s post hoc tests. *P < 0.05, **P < 0.01, ***P < 0.001 compared to WT mice

The gp120tg mice featuring the expression of viral gp120 protein in CNS astrocytes under the control of a modified *GFAP* promoter serve as a surrogate model for HAND [21]. Initially, to determine whether gp120 expression is sufficient to elicit cognitive or emotional impairment, 12-month-old gp120tg mice and age-matched WT mice were subjected to the Morris water maze (MWM) test and open field test. WT mice consistently outperformed gp120tg mice on each training day during the MWM test (Fig. 1A, B). In the probe trial, gp120tg mice showed a concentric swimming pattern within proximity of the wall while spending less time in the targeted quadrant (Fig. 1C, D). Regarding emotionality, gp120tg mice at 12 months of age exhibited decreased dwell in the center of the arena, displaying an anxiety-like behavior in the open field test (Fig. 1E, F). We then performed H&E staining to analyze the histological changes of hippocampal subregions, where gp120tg mice displayed visible neuronal loss (Fig. 1G). Meanwhile, the gp120tg mice exhibited an age-dependent loss of neuronal nuclei (NeuN), microtubule-associated protein 2 (MAP-2), and Synaptophysin in the hippocampus (Fig. 1H), suggesting that gp120 expression causes progressive neuronal destruction in mice.

### Progressive neurotoxic astrocyte activation in gp120tg mice

**Fig. 2:**
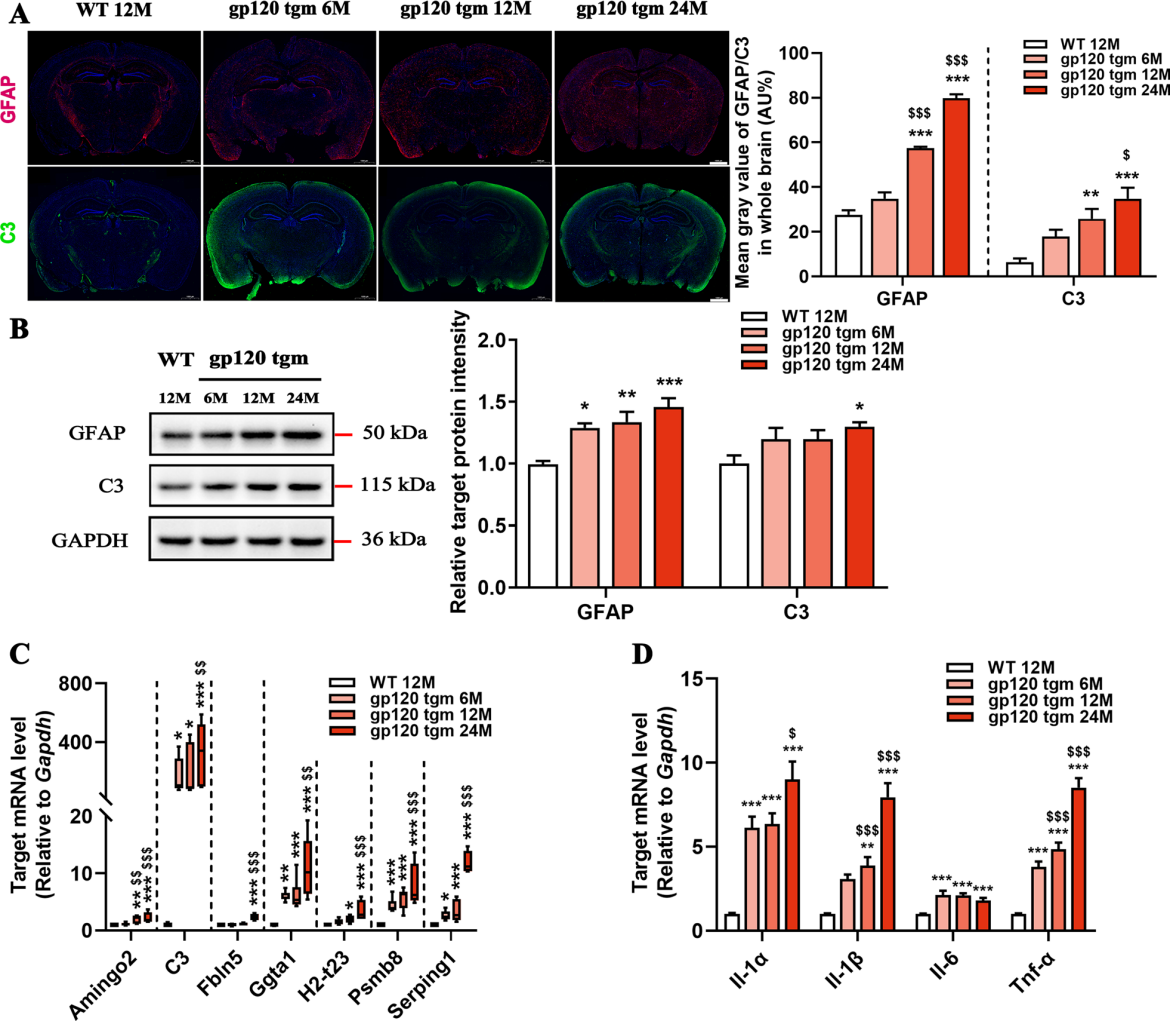
HIV-1 gp120 expression induces progressive A1 astrocyte activation in mice. **A** Immunofluorescence staining against GFAP (red) and C3 (green) in the whole brain sections (scale bar: 1000 μm, *n* = 5 mice per group, *F*_3, 16_ = 147.9, *P* < 0.0001;* F*_3, 16_ = 10.43, *P* = 0.0005, respectively). **B** GFAP and C3 protein levels in the hippocampus of WT and gp120tg mice (*n* = 4 mice per group, *F*_3,12_ = 10.89, *P* = 0.001;* F*_3, 12_ = 3.228, *P* = 0.0609, respectively). The mRNA expression profiles of A1-specific genes (**C**) and cytokines (**D**) in the hippocampus of mice (*n* = 5 mice per group). In **A**, **B**, **D**, data are presented as mean ± SEM. In **C**, values are shown in the box and whisker plot, where the line in the box corresponds to the median. Data are determined by One-way ANOVA with Tukey’s multiple comparisons. *P < 0.05, **P < 0.01, ***P < 0.001 compared to WT mice. ^$^*P* < 0.05, ^$$^*P* < 0.01, ^$$$^*P* < 0.001 compared to 6-month-old gp120tg mice

Glial fibrillary acidic protein (GFAP) and complement component 3 (C3) are markedly enriched in neurotoxic reactive astrocytes from patients with neurodegenerative disorders [8]. To interrogate the effects of gp120 on A1 astrocyte formation, we immunostained brain sections with antibodies against GFAP and C3. Indeed, the expression of GFAP and C3 in the cortex of 6-month-old gp120tg mice was already evident and increased dramatically during the disease progression (Fig. 2A). The immunoblot assay further confirmed elevated GFAP and C3 protein levels in the hippocampus of aged gp120tg mice (Fig. 2B). To better understand how gp120 expression influences the astrocytes in CNS, we analyzed the predominant gene pattern of A1 astrocytes. Strikingly, we observed a significant age-dependent increase in A1-specific genes and proinflammatory cytokines in the hippocampus of gp120tg mice during aging (Fig. 2C, D). Together, our results suggest that gp120-elicited pathological progression in CNS is associated with neurotoxic astrocyte activation.

### α7nAChR knockout rescues cognitive impairment in gp120tg mice via modulation of A1 astrocyte responses

**Fig. 3:**
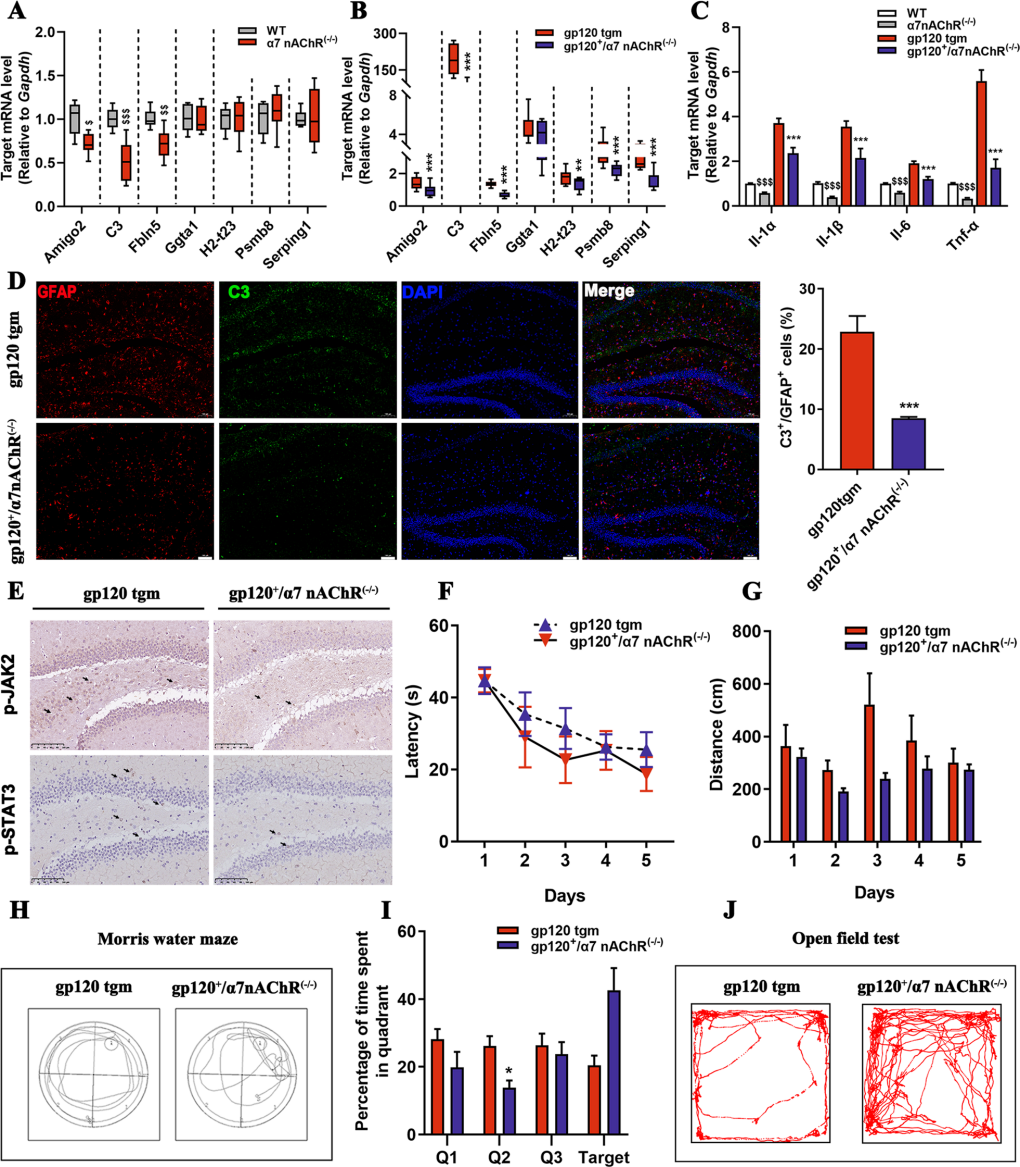
Ablation of the *Chrna7* gene attenuates A1 astrocyte activation and ameliorates cognitive deficits in gp120tg mice. **A** The expression of A1-specific genes in the hippocampus from 12-month-old α7^−/−^ mice and age-matched WT mice. **B** The mRNA levels of A1-specific genes in 12-month-old gp120tg mice and age-matched α7^−/−^gp120tg mice. **C** The mRNA levels of *Il-1α*, *Il-1β*, *Il-6*, *Tnf-α*. **D** The immunoreactivity of GFAP and C3 proteins in the hippocampus (scale bar: 100 μm, *n* = 5 mice per group,* t* = 5.449, *P* = 0.0006). **E** Representative images of immunohistochemistry staining for p-JAK2 and p-STAT3 proteins on brain sections from the hippocampus subregion of the gp120tg mice and α7^−/−^gp120tg mice (scale bar: 100 μm). **F**–**I** The gp120tg mice and α7^−/−^gp120tg mice were subjected to the Morris water maze test (*n* = 10 mice per group). The discrepancy in latency (**F**) and distance (**G**) in acquisition trails (*n* = 6 mice per group, *F*_1, 12_ = 0.7653, *P* = 0.3989; *F*_1, 12_ = 5.341, *P* < 0.0394, respectively). **H** Search strategies for the removed platform in the retrieval test. **I** Percentage of time spent in the different zones in the probe trials (*F*_1, 12_ = 0.5141, *P* = 0.0.4871). **J** Characteristic navigation patterns of mice in the open field test (*n* = 10 mice per group). In **A**, **B**, values are shown in the box and whisker plot, where the line in the box corresponds to the median. In **C**–**I**, data are presented as mean ± SEM. In **A**, **B**, **D**, *P* values are analyzed by unpaired Student’s *t* test (two-tailed). In **C**, *P* values are analyzed by one-way ANOVA followed by Tukey’s multiple comparisons. In **F**–**I**, data are analyzed by repeated measure ANOVA with Bonferroni’s post hoc tests. ^$^*P* < 0.05, ^$$^*P* < 0.01, ^$$$^*P* < 0.001 compared to WT mice. *P < 0.05, **P < 0.01, ***P < 0.001 compared to 12-month-old gp120tg mice

The up-regulation of functional α7nAChR protein was previously found in the gp120tg mice and HIV-infected subjects [22, 23]. To pinpoint the role of α7nAChR in astrocytes, we employed a knockout mice model with a null mutation of the *Chrna7* gene, referred to as α7nAChR knockout mice (α7^−/−^ mice). Downregulation of A1-specific genes and pro-inflammatory cytokines was observed in the hippocampus of α7^−/−^ mice, indicating that the *Chrna7* gene controls A1 astrocyte activation in mice (Fig. 3A, C). To address whether α7nAChR ablation elicits blockage of A1 astrocyte responses in gp120tg mice, we mated the α7^−/−^ mice with gp120tg mice line to generate a crossbreed mice model, α7^−/−^gp120tg mice, which expressed gp120 with concomitant α7nAChR deficiency. As expected, the α7^−/−^gp120tg mice exhibited virtually down-regulated A1-specific genes, which resulted in lower mRNA levels of pro-inflammatory cytokines in α7^−/−^gp120tg mice (Fig. 3B, C). The weak immunoreactivity of GFAP and C3 in the hippocampus further confirmed the downregulation of A1 astrocytes in α7^−/−^gp120tg mice (Fig. 3D). Next, we addressed whether α7nAChR knockout leads to the truncation of JAK2/STAT3 signaling in α7^−/−^gp120tg mice, as STAT3 is highly associated with A1 astrocyte activation [24]. As expected, α7nAChR knockout robustly decreased the expression of JAK2 and STAT3 proteins in the hippocampus of α7^−/−^gp120tg mice (Fig. 3E). Finally, we focused on the cognitive performance of α7^−/−^gp120tg mice. With data collapsed across the 5 training days in the MWM test, α7^−/−^gp120tg mice outperformed gp120tg mice each day during the training session (Fig. 3F, G). The gp120tg mice exhibited wall-hugging behavior in the probe trials, whereas the α7^−/−^gp120tg mice used a focal search strategy for the removed platform showing a clear preference for the target quadrant (Fig. 3H, I). We also observed decreased anxiety-like behavior of the α7^−/−^gp120tg mice in the open-field apparatus (Fig. 3J). Together, our data reinforce that ablation of α7nAChR is necessary and sufficient for inhibiting A1 astrocyte activation, which shields mice from gp120-induced neurotoxicity and cognitive declines.

### KYNA controls A1 astrocyte generation against gp120-induced injury

**Fig. 4:**
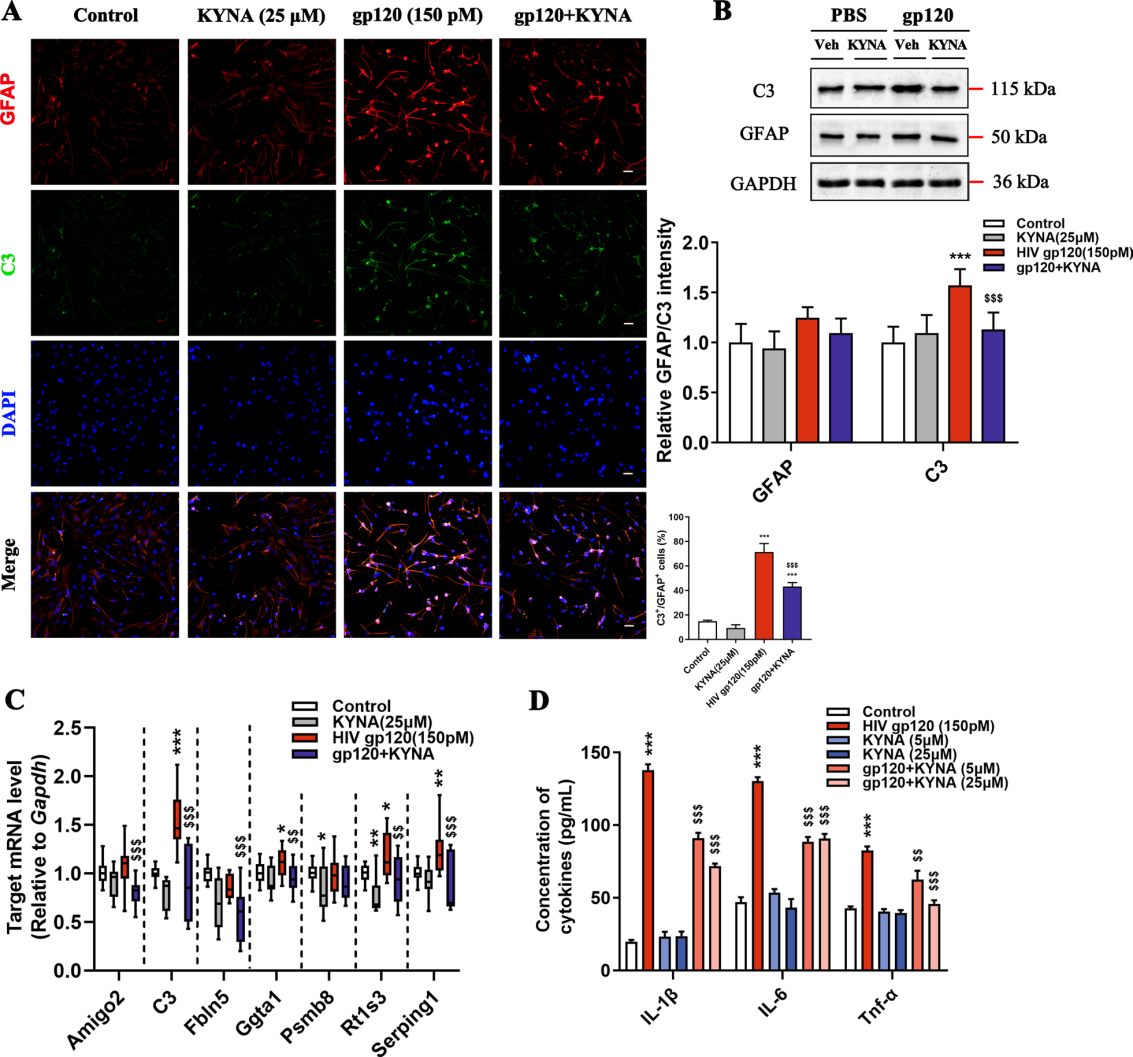
KYNA blunts the gp120-induced A1 astrocyte activation. **A** Double immunofluorescence images showing the expression of GFAP (red), C3 (green), and nucleus (blue) in primary astrocytes (scale bar: 50 μm, *F*_3, 16_ = 49.84, *P* < 0.0001). **B** Quantification of GFAP and C3 protein levels by Western blot (*F*_3, 16_ = 3.734, *P* < 0.033; *F*_3, 16_ = 11.56, *P* = 0.0003). **C** Relative gene expression of A1 phenotype in primary astrocytes. **D** Quantification of Il-1β, Il-6, and Tnf-α levels in cultured supernatant. In **A**, **B**, **D**, data are presented as mean ± SEM. In **C**, values are shown in the box and whisker plot, where the line in the box corresponds to the median. Throughout, *P* values are calculated using one-way ANOVA followed by Tukey’s multiple comparisons with *n* = 5 cultures per group. *P < 0.05, **P < 0.01, ***P < 0.001 compared to control groups. ^$^*P* < 0.05, ^$$^*P* < 0.01, ^$$$^*P* < 0.001 compared to 150 pM HIV-1 gp120 treated group

KYNA, well known as a neuroprotective modulator with α7nAChR inhibitory properties, is mainly synthesized in astrocytes within the CNS [12]. We then determined whether KYNA administration would abrogate gp120-induced A1 astrocyte formation. After the Cy3-conjugated GFAP immunostaining assay, over 98% pure astrocytes culture were incubated with gp120. Consistent with the in vivo findings, sustained gp120 stimulation markedly increased the production of C3 and GFAP in primary rat astrocytes, whereas KYNA dramatically attenuated the immunoreactivity of C3 and GFAP (Fig. 4A, B). Furthermore, After KYNA incubation, primary astrocytes displayed a limited expression of A1-specific genes even under gp120 insult (Fig. 4C). To illuminate the functional changes of KYNA-treated astrocytes, we monitored the cytokine levels in culture. Compared with the gp120 group, KYNA substantially suppressed the release of pro-inflammatory cytokines, with approximately 50% inhibition at 25 μM KYNA concentration (Fig. 4D), suggesting that KYNA attenuates neurotoxic astrocyte responses provoked by gp120.

### KYNA blocks the α7nAChR-mediated JAK2/STAT3 signaling cascade in gp120-induced astrocytes

**Fig. 5:**
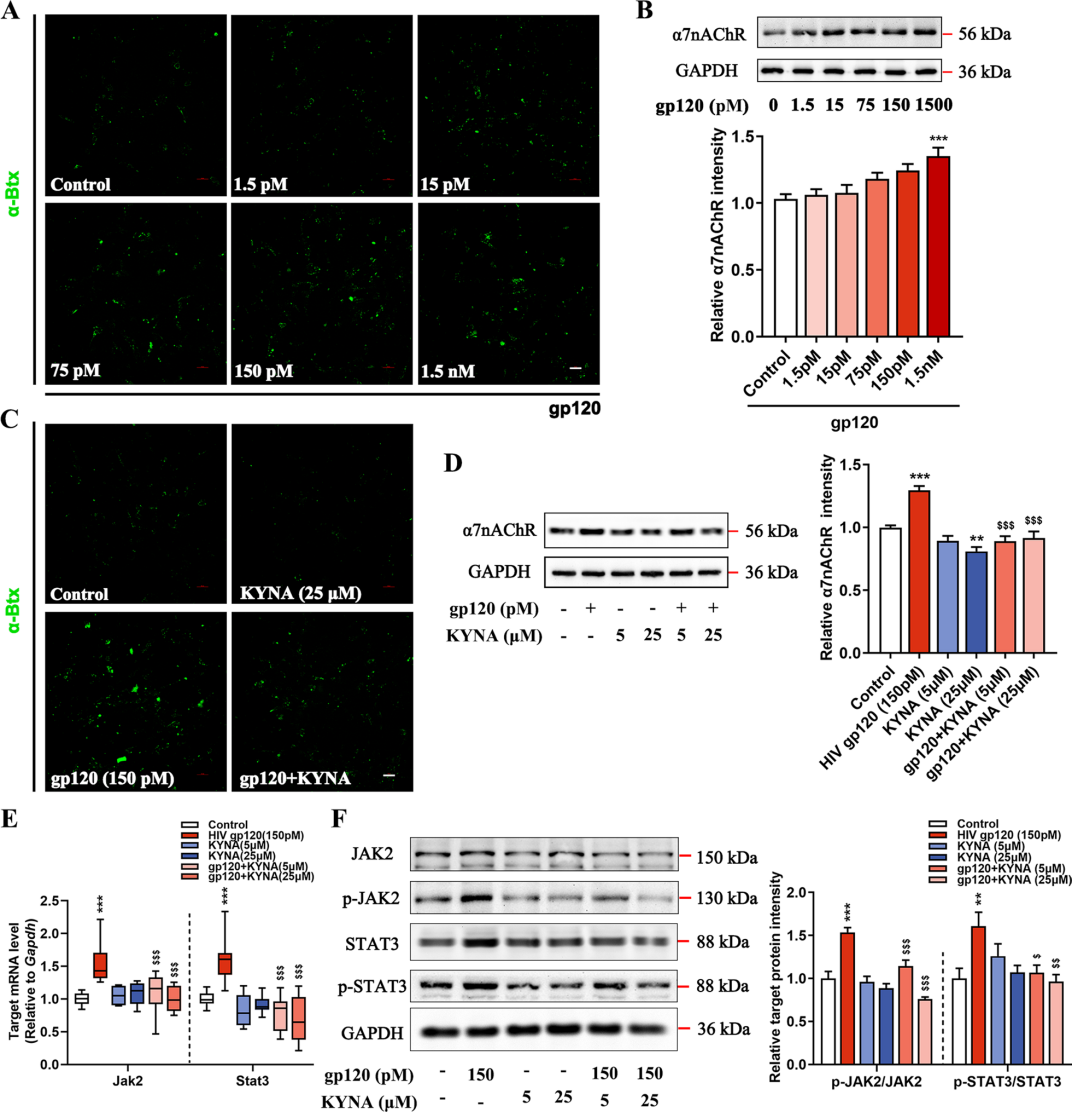
KYNA attenuates the activation of the α7nAChR/JAK2/STAT3 signaling pathway in gp120-induced astrocytes. **A** Astrocytes were treated with gp120 at a wide range of concentrations from 1.5 to 1500 pM for 24 h. The expression of α7nAChR on the cell membrane is indicated by α-bungarotoxin binding images (scale bar: 100 μm). **B** The α7nAChR protein in lysate from astrocytes exposed to increasing doses of gp120 (*F*_5, 42_ = 5.972, *P* = 0.0003). **C** Immunofluorescence showing the effects of KYNA on α7nAChR (scale bar: 100 μm). **D** Densitometric analysis of immunoblot for α7nAChR expression (*F*_5, 114_ = 20.32, *P* < 0.0001). **E** The mRNA levels of *Jak2*/*Stat3*. **F** Protein expression of the JAK2/p-JAK2/STAT3/p-STAT3 relative to the housekeeping protein GAPDH (*F*_5, 60_ = 20.02, *P* < 0.0001; *F*_5, 48_ = 4.311, *P* = 0.0026, respectively). In **B**, **D**, **F**, data are presented as mean ± SEM. In **E**, values are shown in the box and whisker plot, where the line in the box corresponds to the median. Throughout, *P* values are calculated using one-way ANOVA followed by Tukey’s multiple comparisons. *P < 0.05, **P < 0.01, ***P < 0.001. compared to control group. ^$^*P* < 0.05, ^$$^*P* < 0.01, ^$$$^*P* < 0.001 compared to 150 pM HIV-1 gp120 treated group

Since KYNA acts as a negative allosteric regulator of α7nAChR [25], we sought to determine whether α7nAChR expression is altered in gp120-induced astrocytes under KYNA treatment. The enhancement in fluorescence intensity indicates that α7nAChR was abundant in astrocytes following the stimulation with high-dose gp120 (Fig. 5A). Of note, the α7nAChR levels in astrocytes were proportional to gp120 concentration (Fig. 5B), whereas primary astrocytes sparsely expressed α7nAChR on cell membranes after KYNA incubation (Fig. 5C). Later experiments confirmed that KYNA administration resulted in a marked reduction of α7nAChR protein in gp120-induced astrocytes (Fig. 5D). We then addressed whether KYNA blocked JAK2/STAT3 cascade in gp120-infected astrocytes, as JAK2/STAT3 recruitment was previously observed in cells following α7nAChR activation [26]. Indeed, astrocytes showed significant increases in JAK2, STAT3, and phosphorylated counterparts after gp120 infection (Fig. 5E, F). The inhibitory effect of KYNA on JAK2/STAT3 expression in gp120-treated astrocytes was efficient in both mRNA and protein levels (Fig. 5E, F). In general, we suggest that KYNA abolishes the gp120-induced α7nAChR/JAK2/STAT3 signaling activation in astrocytes.

### KYNA attenuates gp120-induced A1 astrocyte formation via blockade of α7nAChR

**Fig. 6:**
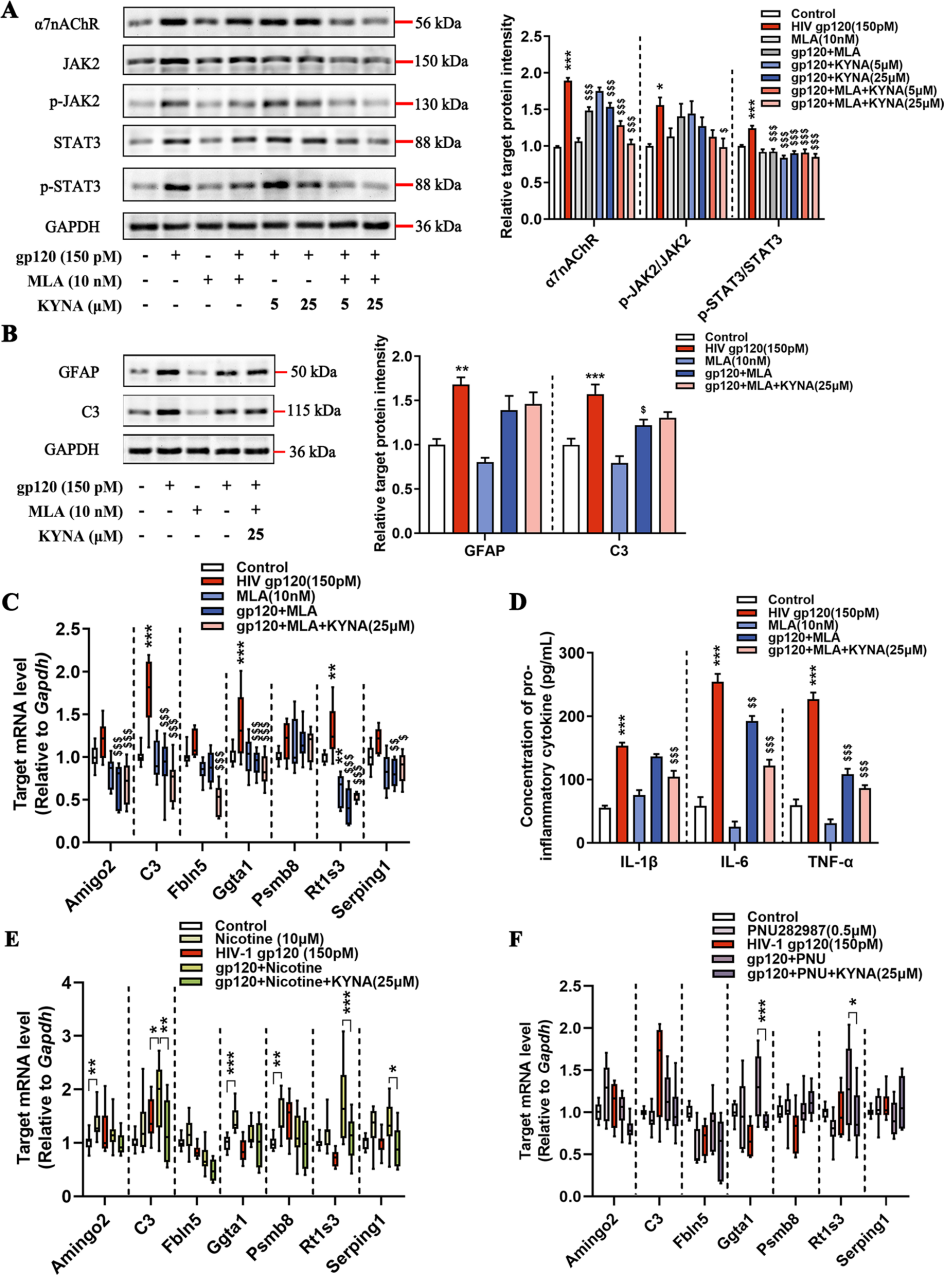
Inhibition of α7nAChR by KYNA and MLA against gp120-induced neurotoxic responses in astrocytes. **A** Protein levels of α7nAChR, JAK2, p-JAK2, STAT3, and p-STAT3 in the astrocytes (*F*_7, 224_ = 5.972, *P* < 0.0001; *F*_7, 72_ = 3.065, *P* = 0.007; *F*_7, 176_ = 12.99, *P* = 0.0003). **B** Immunoblot analysis of the cell lysates presenting the expression of GFAP and C3 proteins (*F*_4, 20_ = 11.50, *P* < 0.0001; *F*_4, 20_ = 14.31, *P* < 0.0001, respectively). **C** Quantification of the expression of A1-specific genes. **D** Protein levels of Il-1β, Il-6, Tnf-α. **E**, **F** Primary rat astrocytes were treated with 150 pM gp120 for 12 h, then pretreated with 10 μM nicotine (**E**) or 0.5 μM PNU-282987 (**F**) for 1 h, followed by co-incubation with 25 μM KYNA for 11 h. The mRNA levels of A1-specific genes were measured. In **A**, **C**, **D**, data are presented as the mean ± SEM. In **B**, **E**, **F**, values are shown in the box and whisker plot, where the line in the box corresponds to the median. Data are analyzed by one-way ANOVA with Tukey’s multiple comparisons. *P < 0.05, **P < 0.01, ***P < 0.001. compared to the control group. ^$^*P* < 0.05, ^$$^*P* < 0.01, ^$$$^*P* < 0.001 compared to 150 pM HIV-1 gp120 treated group

Given the previous results, we postulated that the inhibitory effect of KYNA on A1 astrocyte activation might be attributable to the negative modulation of α7nAChR/JAK2/STAT3 signaling. To investigate this hypothesis, we pre-treated astrocytes with a selective α7nAChR antagonist, methyllycacontitine (MLA). MLA, together with escalating doses of KYNA, pronouncedly reduced the expression of α7nAChR/JAK2/p-JAK2/STAT3/p-STAT3 in gp120-induced astrocytes (Fig. 6A). Interestingly, the decreased expression of GFAP and C3 protein was observed in gp120-induced astrocytes following MLA and KYNA treatment (Fig. 6B). Furthermore, MLA promoted the inhibitory effect of KYNA on the production of A1-specific genes and pro-inflammatory cytokines, indicating that α7nAChR inhibition is essential for controlling A1 astrocyte generation (Fig. 6C, D). Finally, to disentangle the relationship between α7nAChR activation and inhibition in reactive astrocytes, we pre-treated astrocytes with nicotine, a nAChRs activator, or PNU-282987, an α7nAChR agonist. Even though a fraction of A1-specific genes was increased in the astrocytes following nicotine or PNU-282987 pretreatment, KYNA still slightly downregulated A1-specific genes (Fig. 6E, F). Overall, we conclude that KYNA exerts its inhibitory impact on the gp120-induced A1 astrocyte responses via blockage of the α7nAChR/JAK2/STAT3 signaling pathway.

### Inhibition of JAK2/STAT3 signaling suppresses A1 astrocyte activation

**Fig. 7:**
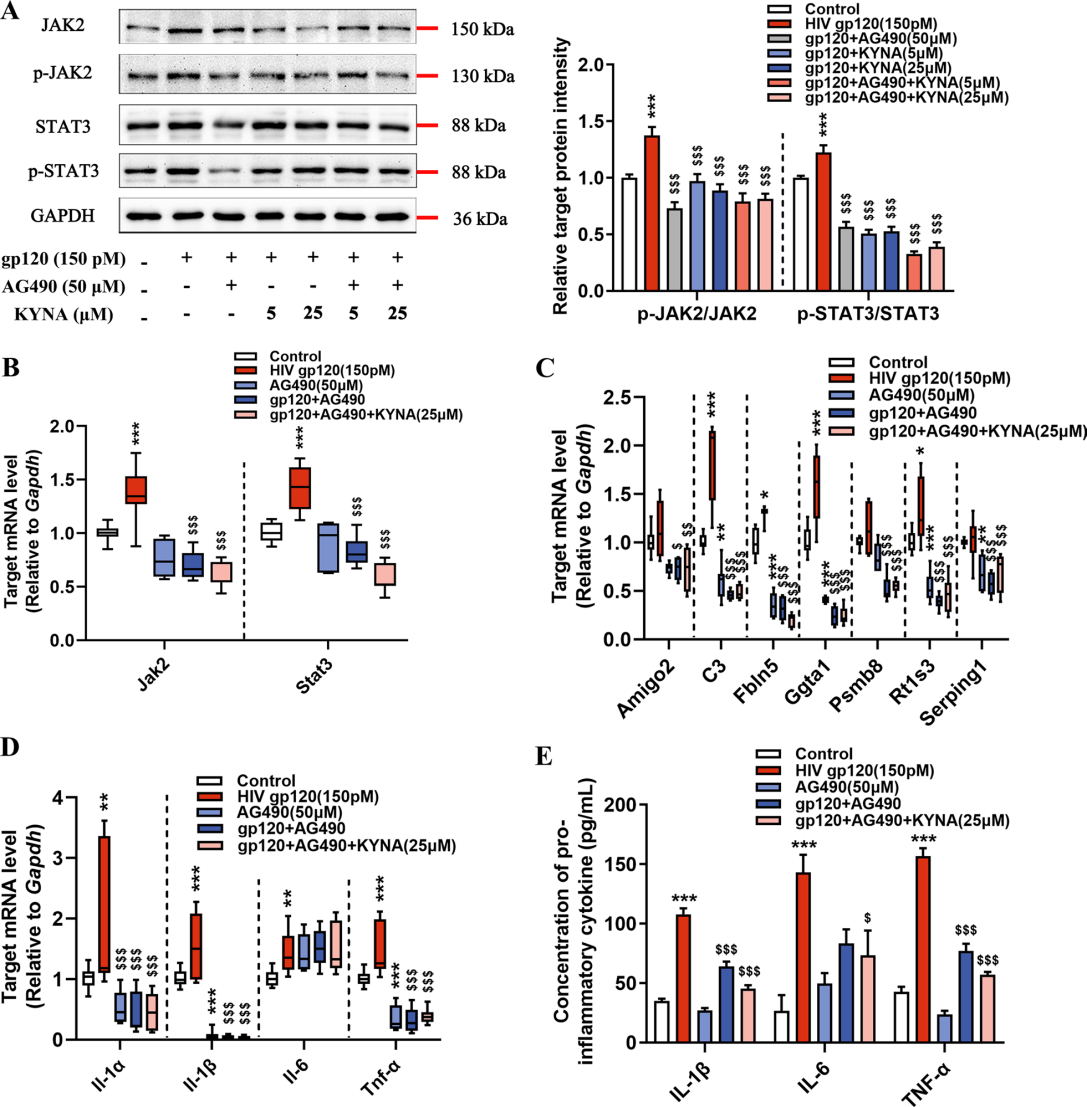
Downregulation of neurotoxic responses in gp120-stimulated astrocytes by targeting the JAK2/STAT3 signaling pathway. **A** Immunoblots showing the protein levels of JAK2/p-JAK2/STAT3/p-STAT3 in stimulated astrocytes (*F*_6, 70_ = 13.95, *P* < 0.0001; *F*_6, 91_ = 69.49, *P* < 0.0001, respectively). **B** The mRNA levels of *Jak2* and *Stat3*. **C** Quantification of A1-specific gene in each treated group. **D** The mRNA levels of *Il-1α*, *Il-1β*, *Il-6*, *Tnf-α* in astrocytes. **E** The protein levels of cytokines in astrocyte culture supernatants as measured by ELISA. In **A**, **E**, data are presented as mean ± SEM. In **B**–**D**, values are shown in the box and whisker plot, where the line in the box corresponds to the median. *P* values are calculated using one-way ANOVA with Tukey’s multiple comparisons. *P < 0.05, **P < 0.01, ***P < 0.001. compared to the control group. ^$^*P* < 0.05, ^$$^*P* < 0.01, ^$$$^*P* < 0.001 compared to 150 pM HIV-1 gp120 treated group

We then asked whether the blockage of A1 astrocyte responses mediated by KYNA is due to the inhibition of STAT3 phosphorylation. We pre-treated astrocytes with AG490, a well-characterized inhibitor for JAK2, to mimic JAK2/STAT3 inhibition via KYNA. Indeed, the lower p-JAK2/JAK2 and p-STAT3/STAT3 ratios were observed in gp120-stimulated astrocytes upon AG490 and KYNA interference (Fig. 7A, B). Furthermore, AG490 and KYNA showed synergy in attenuating the expression of A1-specific genes in gp120-induced astrocytes (Fig. 7C). Subsequent experiments confirmed that the inhibition of JAK2/STAT3 signaling dramatically decreased the transcription and release of pro-inflammatory cytokines in gp120-induced astrocytes (Fig. 7D, E). Collectively, our data suggest that JAK2/STAT3 signaling inhibition is critical for KYNA to blunt the gp120-mediated neurotoxic responses in astrocytes.

### Tryptophan improves cognitive deficits in gp120tg mice by alleviating neuronal damage

**Fig. 8:**
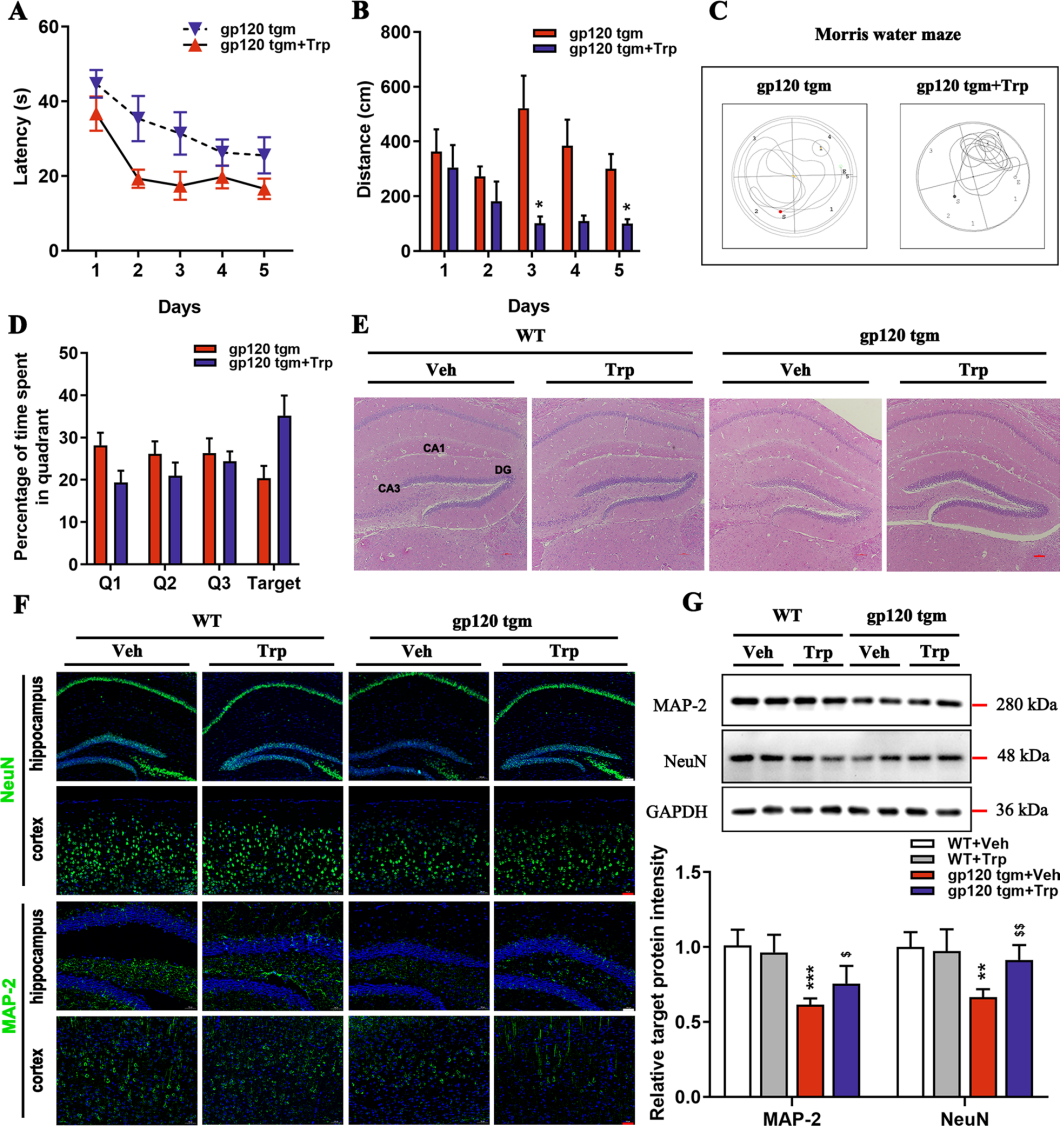
Tryptophan protects against cognitive declines and aberrant neurons in gp120tg mice. **A**–**D** The gp120tg mice were allowed to drink water containing 0.1% tryptophan for 1 month, then were subject to the Morris Water Maze test. **A** Latency to reach the hidden platform and **B** cumulative swim distance between the two groups during the acquisition phase (*n* = 9 mice per group, *F*_1, 15_ = 9.118, *P* = 0.0086; *F*_1, 15_ = 20.08, *P* = 0.0004, respectively). **C** Plots showing the different searching strategies of gp120tg mice and tryptophan-fed gp120tg mice in probe trials. **D** Percentage of time spent in the targeted quadrant in probe trials (*F*_1, 15_ = 1.000, *P* = 0.3331). **E** Brain sections stained with hematoxylin and eosin (scale bar: 100 μm). **F** Immunofluorescence showed the distribution of the NeuN and MAP-2 in the hippocampus (scale bar: 100 μm) and cortex (scale bar: 50 μm) among different groups. **G** The protein levels of MAP-2 and NeuN in the hippocampus (*F*_3, 12_ = 13.57, *P* = 0.0004; *F*_1, 12_ = 8.631, *P* = 0.0025, respectively). Throughout, data present mean ± SEM. In **A**–**D**, *P* values data are analyzed by repeated measure ANOVA with Bonferroni’s post hoc tests. In **G**, data are analyzed by one-way ANOVA with Tukey’s multiple comparisons. *n* = 5 mice per group. *P < 0.05, **P < 0.01, ***P < 0.001 compared to WT mice. ^$^*P* < 0.05, ^$$^*P* < 0.01, ^$$$^*P* < 0.001 compared to 12-month-old gp120tg mice treated with PBS

We designed to reintroduce dietary tryptophan into mice in case to elevate the endogenous KYNA levels in the CNS to investigate what extent tryptophan can alter widespread neuropathology in 12-month-old gp120tg mice. Mice receiving the tryptophan diet spent remarkably shorter time and distance finding the hidden platform than gp120tg mice did on the 1st day onwards, as shown in the acquisition phase of the MWM test (Fig. 8A, B). During the probe trials, tryptophan-fed mice showed focal trajectories targeting the removed platform, resulting in prolonged dwell time in the targeted quadrant (Fig. 8C, D). Next, to address whether cognitive flexibility results from neuronal improvement, we conducted a histological examination of the hippocampus in mice and found lower levels of vacuolization and neuronal loss in the hippocampus of tryptophan-received mice (Fig. 8E). Surprisingly, the tryptophan-fed gp120tg mice showed a significant increase in the expression of NeuN and MAP-2 protein (Fig. 8F, G), suggesting that tryptophan treatment rescues already established neuronal abnormalities in gp120tg mice.

### Tryptophan attenuates neurotoxic astrocyte responses in gp120tg mice

**Fig. 9:**
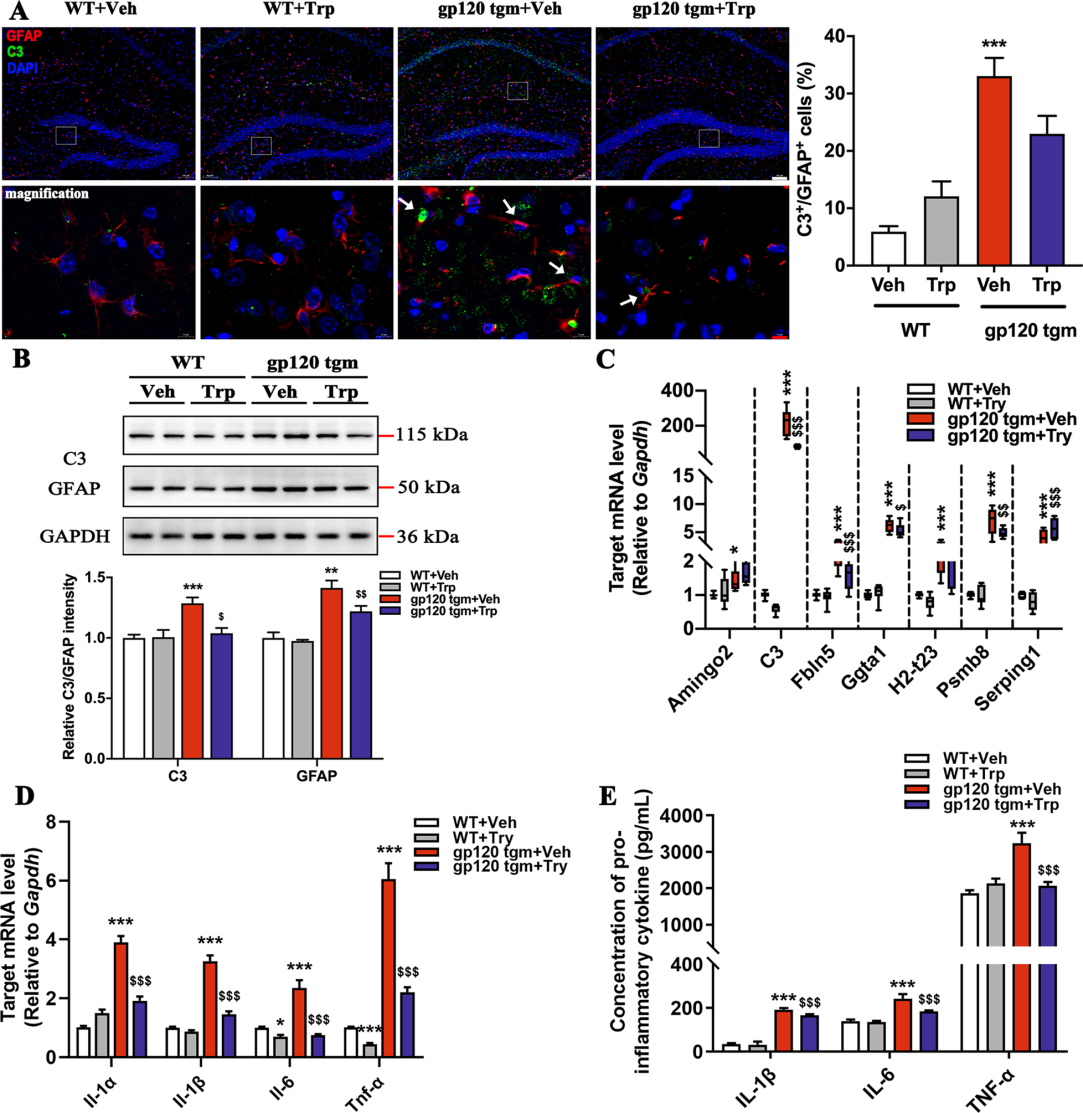
Tryptophan alleviates neurotoxic astrocyte responses in gp120tg mice. **A** Colocalization of GFAP (red) and C3 (green) cells in the hippocampus from mice treated with PBS or tryptophan (scale bar: 100 μm). The white rectangles mark the magnified area. High-magnification images showed co-staining with GFAP and C3 (scale bar: 10 μm). White arrows indicate the GFAP^+^/C3^+^ cells (*F*_3, 16_ = 20.78, *P* < 0.0001). **B** C3 and GFAP protein levels in the brain lysates as determined by Western blot (*F*_1, 12_ = 8.631, *P* = 0.0025; *F*_3, 20_ = 8.846, *P* = 0.0006, respectively). **C** Alterations of A1-specific genes. **D** The mRNA levels of *Il-1α*, *Il-1β*, *Il-6*, and *Tnf-α* in hippocampus. **E** Serum levels of Il-1β, Il-6, and Tnf-α as measured by ELISA. In **A**, **B**, **D**, **E**, data are presented as mean ± SEM. In **C**, values are shown in the box and whisker plot, where the line in the box corresponds to the median. Throughout, data are analyzed by one-way ANOVA with Tukey’s multiple comparisons. *n* = 5 mice per group. *P < 0.05, **P < 0.01, ***P < 0.001 compared to WT mice. ^$^*P* < 0.05, ^$$^*P* < 0.01, ^$$$^*P* < 0.001 compared to 12-month-old gp120tg mice treated with PBS

Consistent with our previous findings, we hypothesized that the neuropathological improvement in tryptophan-fed mice is also associated with the alleviated A1 astrocyte responses. As expected, tryptophan robustly prevented the overactivation of C3^+^/GFAP^+^ cells in the hippocampus of gp120tg mice, leading to decreased C3 and GFAP protein levels in tryptophan-fed gp120tg mice (Fig. 9A, B). Meanwhile, the transcriptomic responses of A1 genes were milder in the hippocampus of gp120tg mice following tryptophan administration, suggesting that tryptophan is as potent as KYNA in suppressing A1 astrocyte activation (Fig. 9C). Finally, to assess whether the tryptophan introduction shapes the inflammatory microenvironment in gp120tg mice, we monitored the cytokine levels of gp120tg mice on the tryptophan diet. Tryptophan counteracted the trend toward higher proinflammatory cytokine expression in mRNA and protein levels in gp120tg mice (Fig. 9D, E). Altogether, our data suggest that tryptophan protects gp120tg mice from neurotoxicity by inhibiting neurotoxic astrocyte activation.

## Discussion

Mechanisms of ongoing neurodegeneration in HIV-infected patients remain elusive, as HIV-1 cannot directly damage neurons, whereas widespread neuronal damage is a pathological hallmark of HAND [27]. In response to injury, quiescent astrocytes undergo pronounced transformations to either a detrimental or neuroprotective phenotype [7]. Subsequent researches on astrocyte heterogeneity indicate that neurotoxic astrocytes aggravate the progression of several neurodegenerative disorders [8, 9, 28]. We found that HIV-1 shed protein gp120 evoked neurotoxic astrocyte activation in young mice without overt neuropathology, indicating that neurotoxic astrocytes precede neuronal injury. Subsequently, neurotoxic reactive astrocytes and neuronal losses were on a dramatic rise causing obvious memory impairment in aged gp120tg mice. This data is reminiscent of previous studies that aging increases the sensitivity of mice to cognitive impairment and executive dysfunction following central gp120 insults due to the prolonged cytokine response within the CNS [29, 30]. Meanwhile, aging induces up-regulation of a cassette of A1-specific genes in mice [31], which is in line with the increased A1-specific gene expressions in aged gp120tg mice. Previous studies have shown that Il-1α, Tnf, and C1q, driven by the activated microglia, promote neurotoxic astrocyte formation [8]. Consequently, Il-1α, Il-1β, and Tnf-α act back to the gp120-stimulated reactive astrocytes, engendering a positive feedback loop that amplifies the neurotoxic response [32]. The levels of pro-inflammatory cytokines in gp120tg mice are higher than in gp120-induced primary astrocytes, partially explaining why the A1 responses are more intense in transgenic mice than in primary astrocytes.

A long-standing question regarding astrocyte activation triggered by sterile inflammatory insults is whether neurotoxic astrocytes can transform into a physiological state. A previous study reported that Mecamylamine, a non-competitive antagonist of nAChR, strongly abolishes nicotine-induced morphological changes in astrocytes, pointing towards blockage of α7nAChR activation facilities the morphological rearrangement in astrocytes [33]. We found that α7nAChR knockout dramatically alleviated neurotoxic astrocyte responses, leading to a pronounced cognitive improvement in gp120tg mice. This encouraged us to utilize the KYNA against cognitive impairment in HAND, as Endogenous KYNA has neuroinhibitory properties attributed to its action as an antagonist of NMDA receptors and α7nAChR [34]. Recently, a study revealed that α7nAChR knockout abrogated the neuroprotective effects of PNU120596, a positive allosteric modulator of α7nAChR, in doxycycline-inducible astrocyte-specific HIV-1 Tat transgenic (iTat) mice, indicating that α7nAChR plays a biphasic role in mouse with HIV-associated neuropathology [35]. To mimic acute neurological AIDS, iTat mice received doxycycline for 7–21 days to trigger the expression of Tat, a crucial protein for HIV-1 gene transcription and replication, in the brain [36]. The opposing effects of α7nAChR on cognitive performance in 12-month-old gp120tg mice compared to 10–14 weeks old iTat mice might, in part, be due to persistent neuroinflammation in the CNS of aging gp120tg mice. Nevertheless, significant reactive astrogliosis was observed in iTat mice [36, 37], whereas α7nAChR knockout robustly downregulated the GFAP expression in the Tat-injured astrocytes and the cortex of the iTat mice, reinforcing that α7nAChR is highly involved in the reactive astrocyte formation [35].

To clarify the downstream events in A1 astrocytes upon KYNA-mediated α7nAChR blockage, we utilize AG490 to pinpoint the JAK2/STAT3 signaling pathway, as STAT3 is highly involved in the formation of reactive astrocytes [38]. AG490 and AG490-KYNA co-incubation downregulates gp120-induced A1 astrocyte formation via JAK2/STAT3 signaling inhibition, although the synergy effect seems negligible. Recent studies have revealed that negative regulation of STAT3 signaling cascades prevents GFAP, a pivotal protein controlling the shape of astrocytes, and C3 protein activation [39–41]. The outcome of STAT3-positive astrocytes to disease is intricate because they are beneficial in the healing process, limiting myelin injury and structural synaptic plasticity [39, 42, 43]. However, they are also associated with blood–brain barrier dysfunction, accumulating β-amyloid levels, neuropathic pain maintenance, iron-activated neuronal apoptosis, and brain metastasis colonization [40, 41, 44–46]. Our findings suggest that STAT3-positive astrocytes exert deleterious effects on gp120tg mice, while KYNA alleviates the neurotoxic responses in astrocytes through blockage of the JAK2/STAT3 signal.

Accumulating evidence based on human studies supports the notion that tryptophan metabolism is deregulated in HIV-infected patients, leading to significant tryptophan depletion in the plasma [47, 48]. Strikingly, supplementation of tryptophan or modulation of tryptophan metabolism ameliorates HIV-1 associated neurotoxicity, but the underlying mechanism remains unknown [49, 50]. KYNA is a promising candidate to control neurodegenerative diseases, as the metabolic alteration associated with increased KYNA levels is neuroprotective in HD flies and Alzheimer’s disease mouse models [51–53]. Meanwhile, a shift towards increased KYNA synthesis is observed in the mammalian brain following tryptophan treatment [13, 54]. For mimicking the elevated levels of KYNA in the CNS of gp120tg mice, we utilize the endogenous synthesis strategies to convert dietary tryptophan to KYNA, as KYNA cannot traffic across the blood–brain barrier due to its polar structure [55]. Furthermore, we demonstrated that tryptophan administration dramatically alleviates A1-astrocyte activation in gp120tg mice, underlying the protective mechanism of tryptophan and its metabolites in neuronal damage and cognitive decline.

In summary, we found that the neurotoxic responses of astrocytes in aged gp120tg mice were intense, whereas inhibition of α7nAChR by KYNA and tryptophan treatment dramatically blunted gp120-induced A1 astrocyte activation. Our findings highlight the protective mechanism of α7nAChR inhibition, which may contribute to the therapeutic interventions for HAND.

## Supplementary Information

**Additional file 1.** Primer for RT-qPCR.

**Additional file 2. **Bands in western blot.

## Data Availability

This study includes no data deposited in external repositories. The data that support the plots within this study are available from the corresponding author upon reasonable request.

## Abbreviations

α7nAChR: α7 Nicotinic acetylcholine receptors
α7^−^^/^^−^ mice: α7NAChR knockout mice
α7^−^^/^^−^gp120tg mice: Gp120 transgenic mice with α7nAChR knockout
C3: Complement component 3
CNS: Central nervous system
ELISA: Enzyme-linked immunosorbent assay
GFAP: Glial fibrillary acidic protein
Gp120: HIV-1 glycoprotein 120
Gp120tg mice: Gp120 transgenic mice
HAND: HIV-associated neurocognitive disorders
JAK2: Janus kinases 2
KYNA: Kynurenic acid
MLA: Methyllycacontitine
MWM: Morris water maze
p-JAK2: Phospho-Janus kinases 2
p-STAT3: Phospho-signal transducer and activator transcription 3
RT-qPCR: Quantitative reverse transcription PCR
SD: Sprague–Dawley
STAT3: Signal transducer and activator transcription 3
Trp: Tryptophan
WT mice: Wild-type
