# Supplementary material for: Kynurenic acid blunts A1 astrocyte activation against neurodegeneration in HIV-associated neurocognitive disorders
Source: J Neuroinflammation. 2023 Mar 30;20:87. doi: 10.1186/s12974-023-02771-4 (PMC10061717; doi:10.1186/s12974-023-02771-4)
Supplement: Supplementary file 1 — Additional file 1. Primer for RT-qPCR. [file 12974_2023_2771_MOESM1_ESM.docx]

**Primer for RT-qPCR**

**Table S1**

| Gene | Forward | Reverse |
| --- | --- | --- |
| GAPDH | TGTGAACGGATTTGGCCGTA | GATGGTGATGGGTTTCCCGT |
| α7nAChR | TGCCAGGACGAGAGTGAGGTG | GGATGCCGATGGTACAGATGATGG |
| JAK2 | AAGTGCGTGCGAGCGAAGATC | ACTGCTGAATGAACCTGCGGAATC |
| STAT3 | AAGCTGACCCAGGTAGTGCT | TCCATGTCAAACGTGAGCGA |
| Il-1a | ATCAGCACCTCACAGCTTCC | TCTCCTCCCGATGAGTAGGC |
| Il-1b | TTGCTTCCAAGCCCTTGACT | GGTCGTCATCATCCCACGAG |
| Il-6 | CACTTCACAAGTCGGAGGCT | TCTGACAGTGCATCATCGCT |
| Tnf | CCTCTCTGCCATCAAGAGCC | AAGTAGACCTGCCCGGACTC |
| Amigo2 | TAAACAGATCCCACCACGCC | GTTGCTTTGGTTCAGTGCGT |
| C3 | TTGTCCCCTTGAAGATCGGC | TCATTCCTTCTGGCACGACC |
| Fbln5 | TGCCAGGATTAAAAAGGATACTCAC | TGCACTGTTGCTGTGCATTC |
| Ggta1 | TTCCATTCGGAGAGGGGGAT | CCCCTTAAAGCACTCCCTGG |
| Psmb8 | CTACGACCTTGCCCGAAGAG | CTCCACTTTCACCCAACCGT |
| RT1S3 | GCGGTTATTGTCAGGAGGCT | TGATGGGCCTCACCAACTTC |
| Serping1 | TCCCATTGAGCACACAGGAC | TGGATGAAGGACTCGTTGGC |

**Note: the origin is Rattus norvegicus.**

**Table S2**

| Gene | Forward | Reverse |
| --- | --- | --- |
| Gapdh | GGAGAGTGTTTCCTCGTCCC | ACTGTGCCGTTGAATTTGCC |
| Amigo2 | TAAACAGATCCCACGCCCAC | GGCCAGGACTGAGAATGGAC |
| C3 | TACTCCCAATGTCCTACGGC | ACTGGCTCCTGTCAACACTG |
| Fbln5 | CACAGCAGCAGTGCACAAAC | AGAGGCCTTGAAATCGTGGG |
| Ggta1 | CCAGTCCCGAGAAGTTCACC | AATCCCCCTCTCCGAATGGA |
| Psmb8 | AAGCACAAGTCAGAGGCAGT | TCAGATCTAGGGTGATGGGTCA |
| RT1S3 | CAAATTGAGATGGCCCACGG | ATGGTGCCAAGCAGGTAAGG |
| Serping1 | TTGTGATCGTGGTACCCGTG | TGAAGATCTGGGTCCTCGGT |
| Il-1a | ACGTCAAGCAACGGGAAGAT | AAGGTGCTGATCTGGGTTGG |
| Il-1b | TGAAATGCCACCTTTTGACAGTG | ATGTGCTGCTGCGAGATTTG |
| Il-6 | GGGACTGATGCTGGTGACAA | AGCATTGGAAATTGGGGTAGGA |
| Tnf | AGCCCACGTCGTAGCAAAC | GATAGCAAATCGGCTGACGG |

**Note: the origin is Mus musculus**
